# Relevance of eosinopenia as an early sepsis marker

**DOI:** 10.1186/cc10168

**Published:** 2011-06-22

**Authors:** EB Moura, MO Maia, JA Araújo Neto, FF Amorim

**Affiliations:** 1 Hospital Santa Luzia, Brasília - DF, Brazil

## Introduction

Early diagnosis of sepsis based on biomarker values has been evaluated. However, there is no ideal marker for this purpose yet.

## Objective

To evaluate eosinopenia as an early sepsis marker.

## Methods

A retrospective study, on a 40-bed surgical-medical intensive care unit (ICU). Data from 300 charts of patients consecutively admitted (between January and March 2009) were collected. The patients were classified as negative (no systemic inflammatory response syndrome (SIRS)), SIRS, sepsis, severe sepsis or septic shock, according to the criteria of the American College of Chest Physicians/Society of Critical Care Medicine. Patients who died or were discharged within 24 hours after admission, with previous hematological disease and those whose data were incomplete were excluded from the study. We compared the eosinophil cell count (hematology analyzer ABX Pentra DF 120; Horiba Medical, Montpellier, France) on the day of admission to the ICU between the non-infected group (negative and SIRS) and the infected group (sepsis, severe sepsis and septic shock). The normality of the distribution was tested by the Kolmogorov-Smirnov test and the comparisons were made utilizing the Mann-Whitney test. Statistical analyses were done utilizing SPSS 19 version.

## Results

**Figure 1:**
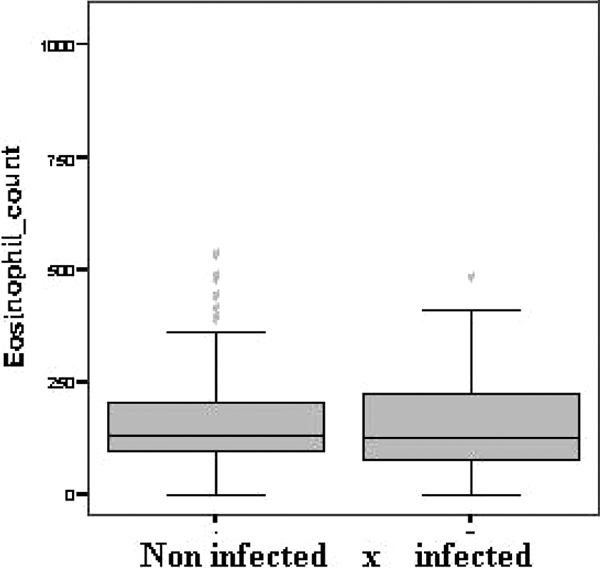
**Eosinophil count comparison between non-infected and infected patients**.

Three hundred patients were admitted to the ICU in the period, mean age 58.6 ± 20 years. The mean length of stay was 9.2 ± 15.7 days, the mean APACHE II score was 9.4 ± 6.5. Eighteen patients were excluded (one because of discharge within 24 hours; 11 patients because of previous hematological disease; six because of incomplete data). The remaining 282 patients were enrolled into the study, classified as follows: negative (158 patients - 56%), SIRS (25 - 8.8%), sepsis (44 - 15.6%), severe sepsis (23 - 8.2%) and septic shock (32 - 11.4%). At the time of admission, 99 (35.1%) patients had an infection. The mean ± SD eosinophil count was 167.6 ± 131.5, 153.6 ± 129 and 153.7 ± 135.6 cells/mm^3 ^in the total, non-infected and infected groups, respectively (*P *= 0.46; Figure 1). At a cut-off value of 100 cells/mm^3^, the eosinophil count yielded a sensitivity of 35%, a specificity of 71%, a PPV of 40% and a NPV of 66%.

## Conclusion

Eosinopenia was not a good early diagnostic marker for sepsis in this population.

